# Hit identification of IKKβ natural product inhibitor

**DOI:** 10.1186/2050-6511-14-3

**Published:** 2013-01-07

**Authors:** Chung-Hang Leung, Daniel Shiu-Hin Chan, Ying-Wei Li, Wang-Fun Fong, Dik-Lung Ma

**Affiliations:** 1 State Key Laboratory of Quality Research in Chinese Medicine, Institute of Chinese Medical Sciences, University of Macau, Macao, China; 2 Department of Chemistry, Hong Kong Baptist University, Kowloon Tong, Hong Kong; 3 Centre for Cancer and Inflammation Research, School of Chinese Medicine, Hong Kong Baptist University, Kowloon Tong, Hong Kong

## Abstract

**Background:**

The nuclear factor-κB (NF-κB) proteins are a small group of heterodimeric transcription factors that play an important role in regulating the inflammatory, immune, and apoptotic responses. NF-κB activity is suppressed by association with the inhibitor IκB. Aberrant NF-κB signaling activity has been associated with the development of cancer, chronic inflammatory diseases and auto-immune diseases. The IKK protein complex is comprised of IKKα, IKKβ and NEMO subunits, with IKKβ thought to play the dominant role in modulating NF-κB activity. Therefore, the discovery of new IKKβ inhibitors may offer new therapeutic options for the treatment of cancer and inflammatory diseases.

**Results:**

A structure-based molecular docking approach has been employed to discover novel IKKβ inhibitors from a natural product library of over 90,000 compounds. Preliminary screening of the 12 highest-scoring compounds using a luciferase reporter assay identified 4 promising candidates for further biological study. Among these, the benzoic acid derivative (**1**) showed the most promising activity at inhibiting IKKβ phosphorylation and TNF-α-induced NF-κB signaling *in vitro*.

**Conclusions:**

In this study, we have successfully identified a benzoic acid derivative (**1**) as a novel IKKβ inhibitor via high-throughput molecular docking. Compound **1** was able to inhibit IKKβ phosphorylation activity *in vitro*, and block IκBα protein degradation and subsequent NF-κB activation in human cells. Further *in silico* optimization of the compound is currently being conducted in order to generate more potent analogues for biological tests.

## Background

The nuclear factor-κB (NF-κB) proteins are a small group of heterodimeric transcription factors that play an important role in regulating inflammatory, immune, and apoptotic responses [1-3]. NF-κB is ubiquitously present in the cytoplasm and its activity is normally suppressed by association with inhibitor IκB [4]. The intracellular NF-κB signaling cascade is initiated by a variety of inducers including proinflammatory cytokines TNF-α, IL-1 or endotoxins [5,6]. The aberrant activity to the NF-κB signaling pathway has been implicated in the development of a number of human diseases including cancer, auto-immune and chronic inflammatory conditions [3,7,8]. Therefore, inhibitors of the NF-κB signaling pathway could offer potential therapeutic value for the treatment of such diseases [9,10].

The IκB kinase is a multi-component complex composed of two catalytic subunits, IKKα and IKKβ and a regulatory unit NF-κB essential modulator (NEMO) [11-13]. Although both catalytic units are able to phosphorylate IκB, IKKβ has been shown to play the dominant role in activating NF-κB signaling in response to inflammatory stimuli [14,15]. Phosphorylated IκB is subsequently tagged by the E1 ubiquitin enzyme and degraded by the proteasome to liberate active NF-κB. Free NF-κB then translocates into the nucleus, where it binds to its cognate DNA site and enhances the expression of a number of genes related to the immune response, cell proliferation and survival [16,17]. Consequently, IKKβ represents an attractive target in the NF-κB pathway for the development of anti-inflammatory or anti-cancer therapeutics.

Virtual screening (VS) has emerged as a powerful tool in drug discovery complementing the vast array of popular but relatively costly high-throughput screening technologies [18,19]. Using virtual screening, the number of compounds to be evaluated *in vitro* could be dramatically decreased, which could greatly reduce the time and resource costs of drug discovery efforts. Meanwhile, natural products (NPs) have long provided a valuable source of inspiration to medicinal chemists due to the diversity of their molecular scaffolds, favourable biocompatibility and evolutionarily validated bioactive substructures [20,21]. Combining these two ideas, our group has previously identified natural product or small molecule inhibitors antagonizing cancer or inflammation-related targets using virtual screening [22-28]. For example, we have successfully identified natural product or natural product-like compounds targeting the c-*myc* oncogene G-quadruplex, tumor necrosis factor-alpha (TNF-α) and NEDD8-activating enzyme (NAE) [29-34].

**Figure 1:**
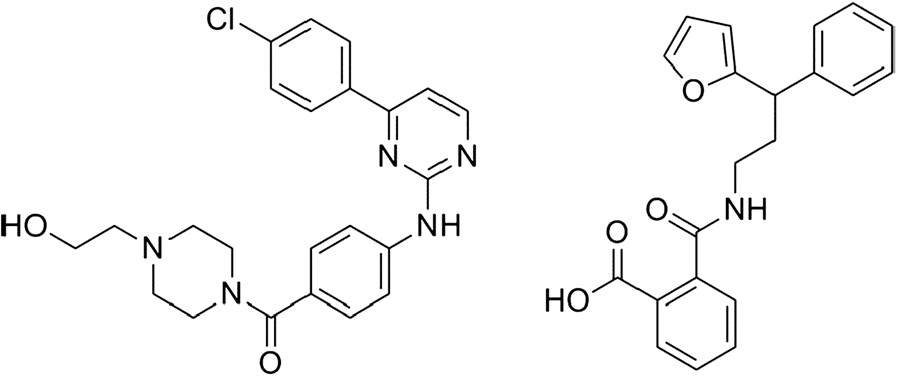
**Chemical structures of the small molecule IKKβ inhibitors.** Chemical structures of IKKβ inhibitors (4-{[4-(4-chlorophenyl)phyrimidin2-yl]amino}phenyl[4-(2-hydroxyethyl)piperazin-1-yl]methanone (reference compound) (left) and NP-derived benzoic acid derivative (**1**) (right).

In recent years, many small molecule inhibitors of IKKβ have been identified using pharmacophore-based or high-throughput screening approaches [32-39]. However, the recent publication of the IKKβ X-ray crystal structure with its inhibitor [40] enables the use of powerful structure-based *in silico* methods for the discovery of novel IKKβ inhibitors. We thus set out to identify interesting molecular scaffolds for the development of future IKKβ inhibitors from a large natural product library using high-throughput structure-based virtual screening. The X-ray co-crystal structure of the IKKβ with the reference inhibitor ((4-{[4-4-chlorophenyl)pyrimidin-2-yl]amino}phenyl[4-(2-hydroxyethyl)piperazin-1-yl]methanone (PDB: 3RZF) was used for our molecular modeling investigations (Figure 1) [40]. To our knowledge, this work is the first example of an IKKβ inhibitor identified using high-throughput molecular docking of a natu-ral product database against the IKKβ X-ray co-crystal structure.

## Results and Discussion

### High-throughput virtual screening

**Scheme 1:**
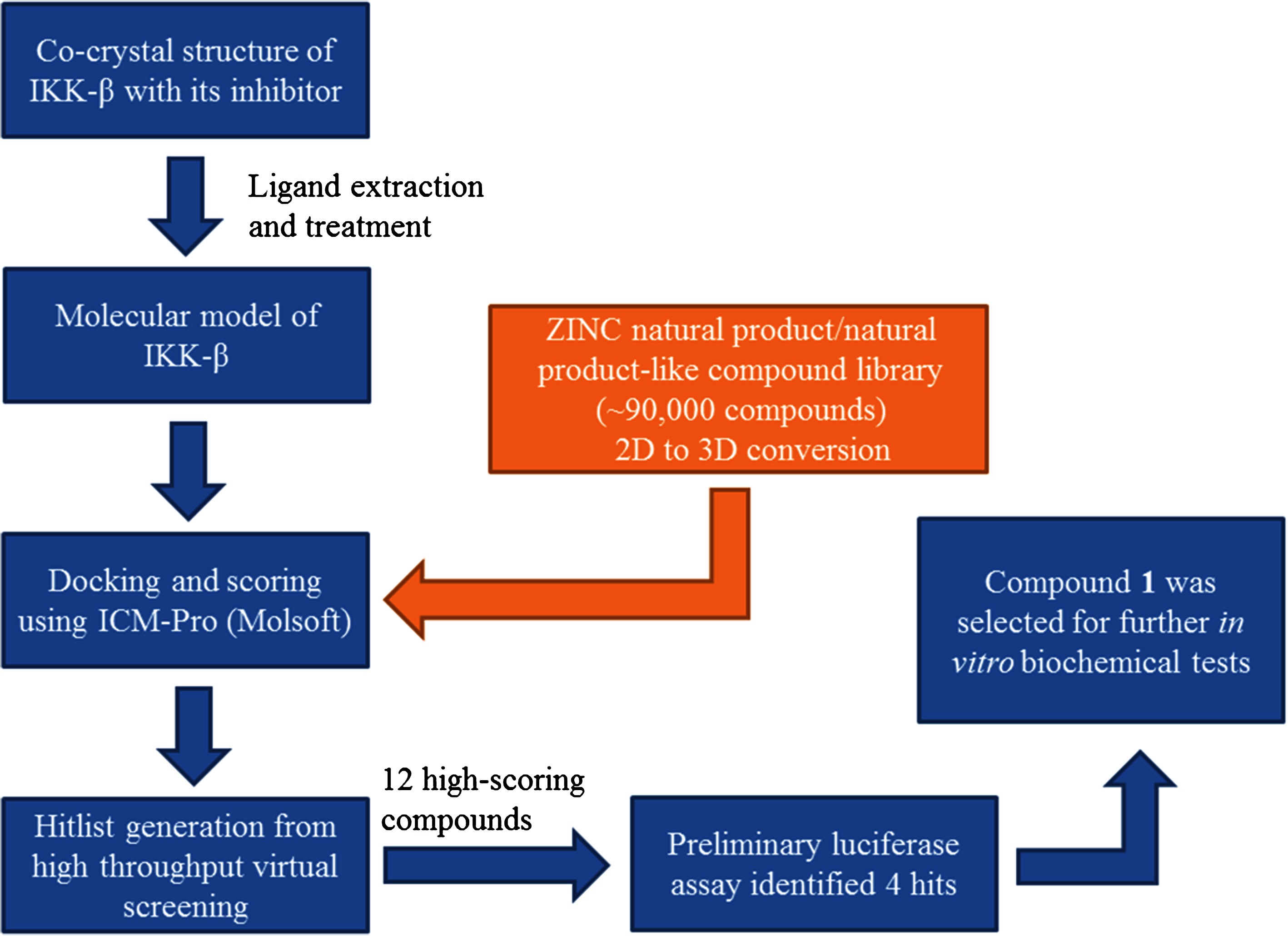
Schematic diagram showing the workflow of this high-throughput molecular docking campaign.

The workflow of this virtual screening (VS) campaign is outlined in Scheme 1. The molecular model of IKKβ for VS was built using the recently reported X-ray co-crystal structure of IKKβ with its inhibitor. The binding site of IKKβ was defined to be within 3Å of the bound inhibitor, which is situated at the hinge loop connecting the N and C lobes of the IKKβ KD domain. Over 90,000 structures from a chemical library of natural products and natural product-like compounds were screened *in silico* against the binding pocket of IKKβ [31]. The flexible ligands were docked to a grid representation of the receptor and assigned a score reflecting the quality of the complex according to the Internal Coordinate Mechanics (ICM) method [ICM-Pro 3.6-1d molecular docking software (Molsoft)]. Compounds with ICM docking scores of under -30 kcal/mol were shortlisted. Based on visual inspection and availability from the commercial sources, 12 compounds containing distinctive chemical scaffolds were chosen (Additional file 1: Figure S1). These compounds were purchased and were subjected to a preliminary luciferase assay (Additional file 1: Figure S2). The results showed that 4 out of the 12 compounds were able to inhibit NF-κB transcription activity by 20% or more compared to the untreated control at a concentration of 20 μM. The benzoic acid derivative (**1**) (Figure 1) inhibited NF-κB activity by over 40% relative to the untreated control, while compounds **3**, **9** and **10** exhibited weaker inhibitory activities of 20–30%. Compounds containing the benzoic acid moiety are known to display a variety of pharmacological effects and a number of benzoic acid derivatives possessing anti-inflammatory properties have been isolated from natural sources. For example, (*E*)-3-acetyl-6-(3,7-dimethylocta-2,6-dienyloxy)-2,4-dihydroxybenzoic acid isolated from *M. semecarpifolia* has been reported to suppress fmet-Leu-Phe (fMLP)-induced superoxide anion generation and elastase release by human neutrophils (Additional file 1: Table S1) [41]. In addition, the natural benzenoid antrocamphin A extracted from the fruiting body of *A. camphorata* was found to down-regulate iNOS and COX-2 expression at both transcriptional and translational levels *via* suppression of NF-κB nuclear translocation [42]. A synthetic benzoic acid-derived compound GS143 reported by Furuichi, Shimbara and co-workers blocked NF-κB translocation through inhibition of IκBα ubiquitination and subsequent IκBα degradation [43]. To our knowledge, no biological activity of **1** has been reported in the literature. The identification of this natural product-derived benzoic acid scaffold as an IKKβ inhibitor could contribute to an understanding of the molecular mechanisms of the anti-inflammatory properties of this class of compounds. Furthermore, we envisage that this natural product derivative could serve as a valuable scaffold for the development of future IKKβ inhibitors.

### Molecular modeling analysis

**Figure 2:**
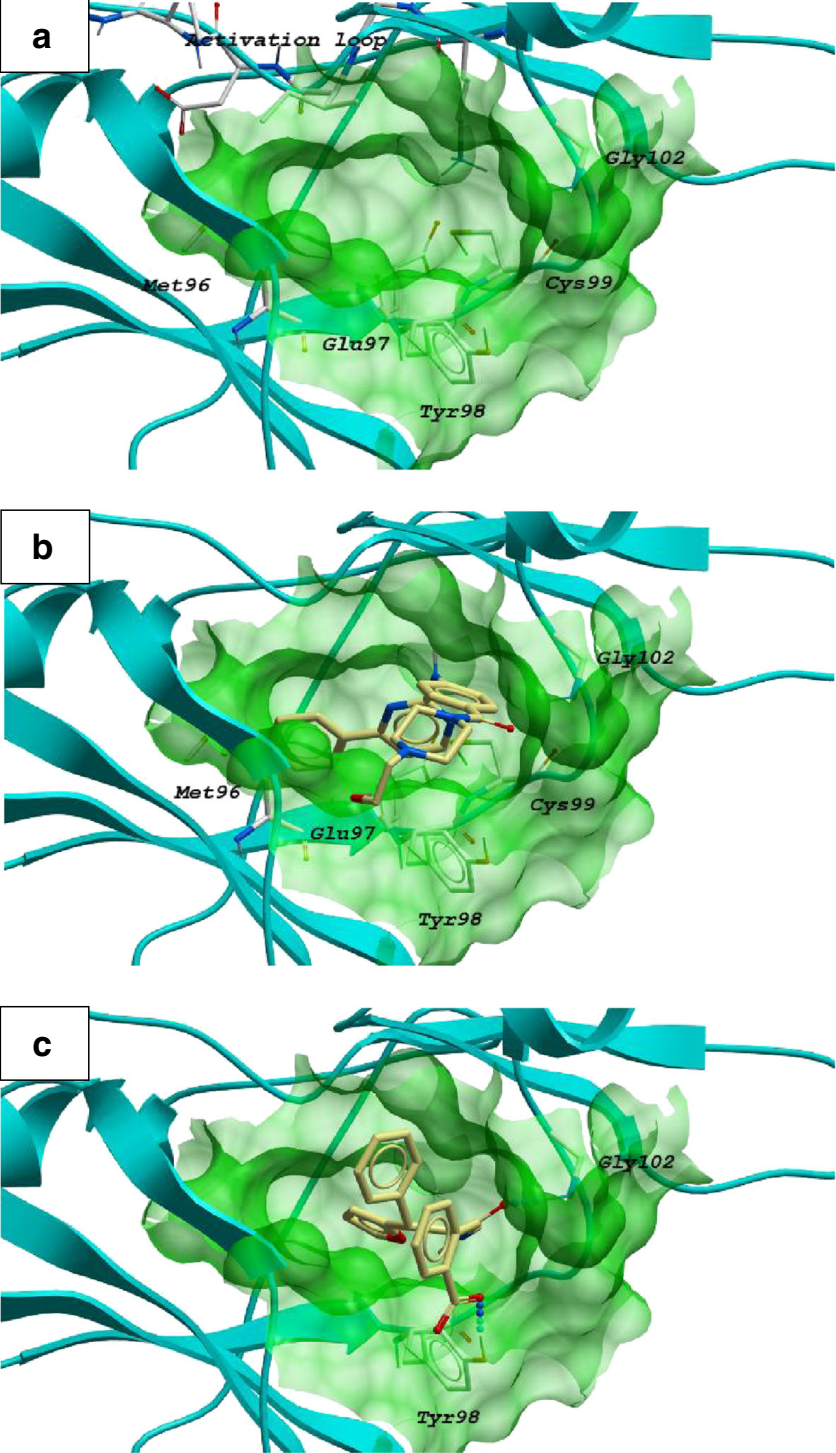
**Molecular docking analysis of reference compound and 1 to the IKKβ protein complex. a**) The KD domain of IKKβ is displayed in the ribbon form. The activation loop, gatekeeper residue (Met 96), hinge region (Glu97-Cys99) are visualized. Low-energy binding conformations of **b**) **1** and **c**) reference compound to IKKβ protein complex were generated by virtual ligand docking. Small molecules inhibitor **1** and reference compound is depicted as a ball-and-stick model showing carbon (yellow), hydrogen (grey), oxygen (red) and nitrogen (blue) atoms. H-bonds are indicated as dotted lines. The binding pocket of the IKKβ is represented as a translucent green surface.

The ATP binding site of kinases generally consists of a narrow and hydrophobic region located between the N-lobe and C-lobe of the kinase domain (KD), with the two lobes linked together by a hinge region consisting of hydrogen bond donor and acceptor residues from the protein backbone [44]. The most important receptor residue in determining kinase inhibitor specificity is the “gatekeeper” residue, which controls the access of the inhibitor to the hydrophobic pocket. In the crystal structure of IKKβ, the gatekeeper residue is Met96, while Glu97, Tyr98 and Cys99 form the hinge region of the KD of IKKβ. The backbone groups of Glu97 and Cys99 are able to provide hydrogen bonding interactions with the inhibitor. In addition, the ATP binding site of IKKβ is partly covered by an activation loop comprised of serine, threonine and tyrosine residues in the unphosphorylated state. In particular, the N-terminal side of the activation loop contains the Asp166, Leu167 and Gly168 DLG triad which is involved in catalytic transfer of the γ-phosphate group in most kinase ATP binding sites (Figure 2a) [40].

Our molecular docking analysis revealed that the top-scoring binding mode of the natural product derivative **1** to the IKKβ complex is similar to that of the reference compound. The bound inhibitor in the co-crystal structure of IKKβ interacts with the ATP binding pocket in shape-driven manner [40]. While the structure of the reference compound contains the anilinopyrimidine motif that is found in other kinase inhibitors such as imatinib [45], no detectable hydrogen bonds between the hinge region of IKKβ and the anilinopyrimidine moiety of the reference compound were recorded. The aromatic rings of the reference compound span the hinge loop while its terminal chlorine atom points towards the gatekeeper residue Met96 (Figure 2b).

By comparison, the benzoic acid moiety of **1** is situated at the end of the hinge loop with predicted hydrogen bonding interactions between the carboxyl oxygen and amide oxygen atoms of **1** with the phenolic hydrogen atom of Tyr98 and the backbone amino group of Gly102, respectively (Figure 2c). The pendant side chain of **1** is predicted to be situated in a hydrophobic binding pocket also occupied by the reference compound. We envisage that **1** could act as a reversible inhibitor of IKKβ by blocking the nucleotide recognition domain that binds ATP [40]. The binding score for **1** with the IKKβ complex was calculated to be -35.28 kcal/mol, reflecting a strong interaction between the compound and the IKKβ binding site.

The other eleven compounds were also predicted to situate in the hinge region of the binding pockets in the docking analysis. Most of the compounds could form hydrogen bonds with the hinge residues including Glu97, Cys99 and Glu100. Furthermore, several of the compounds formed additional hydrogen bonds with the residues in the solvent accessible region (Arg31 and Lys106). The lowest energy binding pose of the other compounds are summarized in Additional file 1: Table S2.

We also investigated the selectivity of compound **1** for IKKβ over four other kinases (PKCα, PAK4, CaMK2α and JAK2) using molecular modeling. While compound **1** was predicted to bind at the ATP binding sites of the four other kinases, the ICM docking energies of the **1**-kinase complexes were significantly less negative than that for IKKβ (Additional file 1: Table S3). Molecules exhibiting such weak binding energies would be expected to be inactive *in vitro*.

### 1 inhibits IκBα phosphorylation *in vitro*

**Figure 3:**
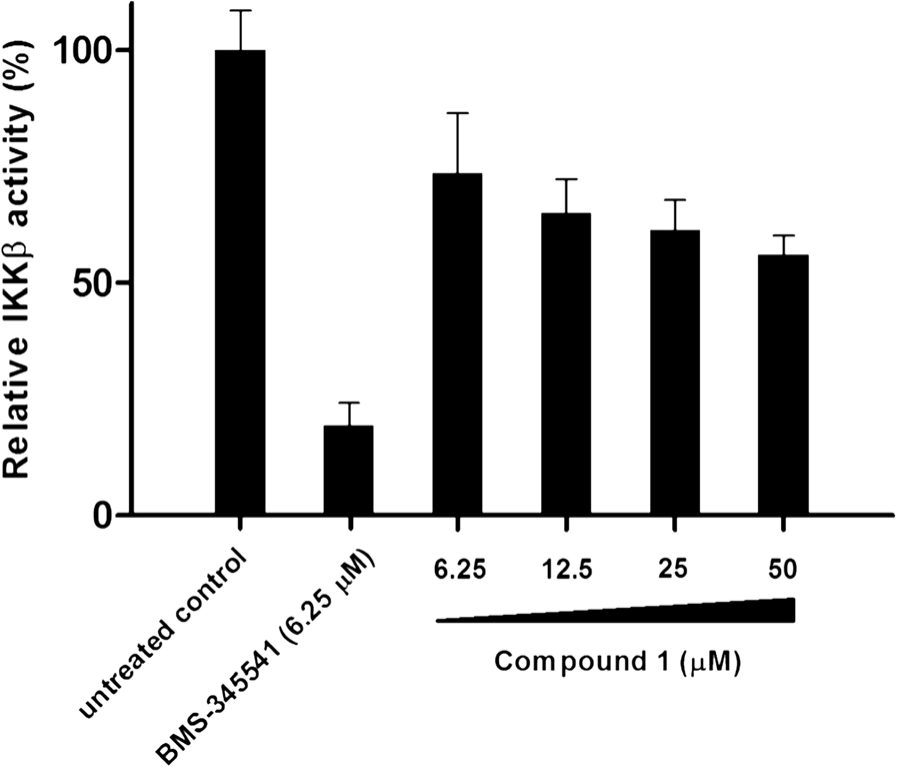
**Inhibition of IKKβ phosphorylation activity.** Microtiter plates with GST-IκBα were incubated with IKKβ together with **1** at the indicated concentrations. GST-IκBα phosphorylated level was detected using an anti-phospho IκBα (Ser32/Ser36) antibody and horseradish peroxidase conjugated secondary antibody. Approximate IC_50_ value of **1** = 50 μM. Error bars represent the standard deviations of the results from three independent experiments.

Encouraged by the molecular docking results and the preliminary luciferase screening experiment, we investigated the effect of compound **1** on IKKβ phosphorylation activity. Inhibition of IKKβ phosphorylation activity would be expected to lead to a decrease in GST-IκBα substrate phosphorylation level. Encouragingly, a dose-dependent reduction in IKKβ activity was observed upon the incubation with **1**, with an estimated IC_50_ value of *ca.* 50 μM (Figure 3).

### 1 inhibits TNF-α induced NF-κB signaling in a HepG2 cell line

**Figure 4:**
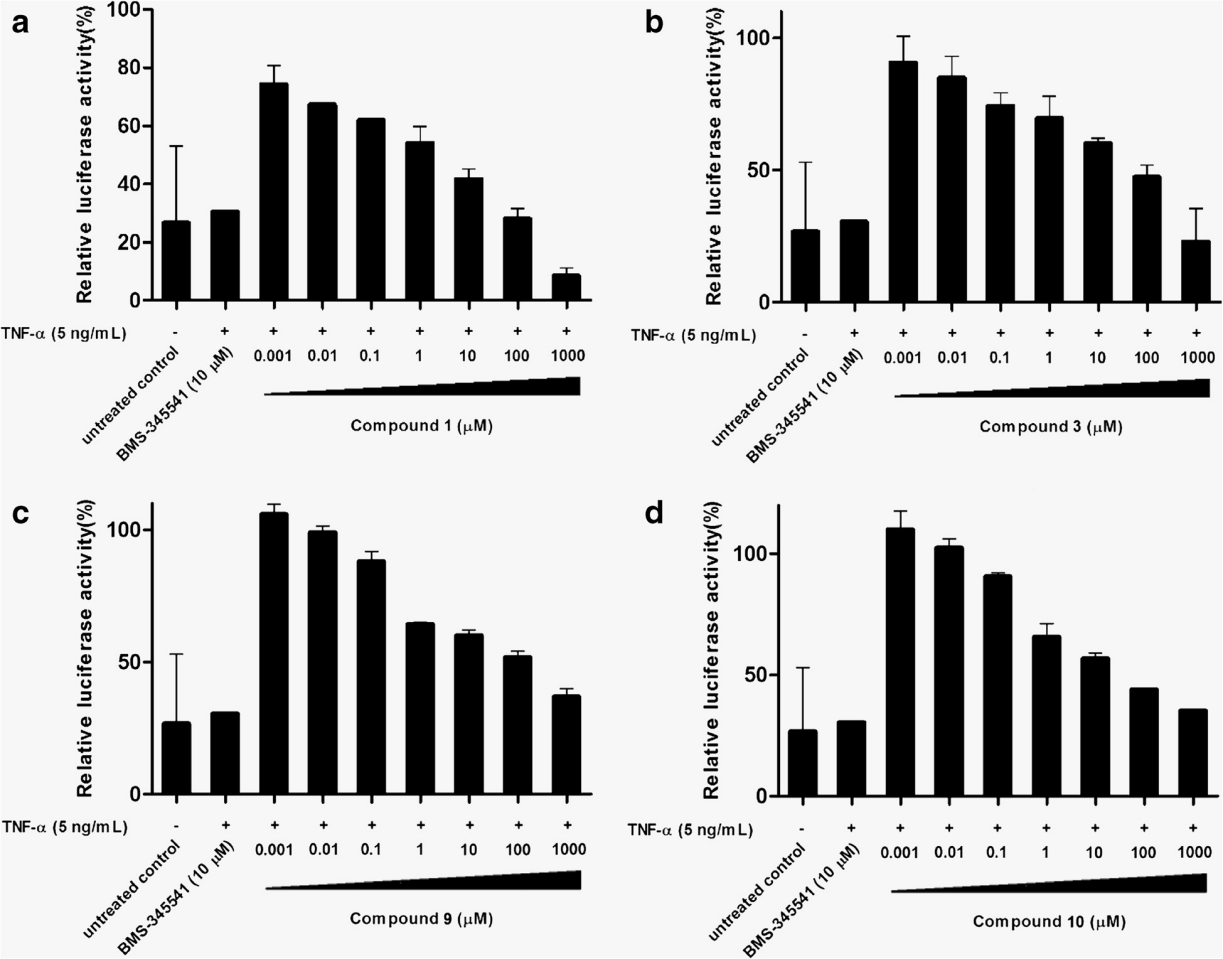
**Inhibition of cellular IKKβ mediated NF-κB activity.** HepG2 cells stably transfected with the NF-κB–luciferase gene were stimulated with TNF-α pre-incubated with the indicated concentrations of **1** and other three hit compounds (compounds **3**, **9** and **10**). Cell lysates were analyzed for luciferase activity to determine the extent of NF-κB inhibition. Error bars represent the standard deviations of the results from three independent experiments.

We sought to investigate the ability of compound **1** to inhibit NF-κB signaling in human cells using a luciferase assay. A stably-transfected HepG2 cell line carrying the luciferase reporter gene driven by a promoter containing multiple copies of the NF-κB response element was used in this study. The transcriptional activity of NF-κB was determined by measuring the luciferase activity of the cell lysates using a luminometer. We performed a dose response analysis of compound **1** and three other hit compounds in attenuating TNF-α-induced NF-κB signaling (Figure 4). Compound **1** inhibited TNF-α-induced luciferase activity in a dose-dependent manner with an estimated IC_50_ value of *ca.* 10 μM. While the three other compounds also inhibited TNF-α-induced luciferase activity, their inhibition potencies were around 10-fold lower compared to compound **1**.

Based on the results of the IKKβ assay and the molecular modeling analysis, we envisage that the inhibition of TNF-α-induced NF-κB signaling by **1** could be attributed, at least in part, to the inhibition of IKKβ activity *in vitro*, thus preventing the degradation of the NF-κB repressor IκBα. The slightly higher potency of **1** in the cell-based luciferase assay compared to the enzyme assay is possibly due to a multi-target effect of **1**, suggesting that this compound could potentially influence other steps involved in NF-κB activation.

## Conclusions

In conclusion, we have discovered a new small molecule IKKβ inhibitor from a large natural product library of 90,000 compounds using high-throughput structure-based molecular docking. The benzoic acid derivative **1** is able to inhibit IKKβ activity in both cell-free and system with micromolar potency. Furthermore, compound **1** could inhibit IKKβ-mediated NF-κB signaling pathway in human cancer cells. We envisage that compound **1** attenuates the *in cellulo* transcriptional activity of NF-κB, at least in part, by abrogating the activity of IKKβ. The discovery of this natural product-like derivative provides medicinal chemists with a structurally interesting scaffold, facilitating further chemical modifications in order to sample greater regions of the chemical space of potential IKKβ inhibitors. We are currently investigating the effects of **1** on the proteins involved in NF-κB signaling and conducting *in silico* lead optimization to generate more potent analogues of **1** for *in vitro* biological testing.

## Methods

### Materials and cell lines

The NP/NP-like compound collection, which includes compound **1** and the other tested compounds, was obtained from InterBioScreen (Moscow, RUS). The K-LISA™ IKKβ Inhibitor Screening Kit was obtained from Calbiochem (Darmstadt, Germany). Passive lysis buffer and luciferase assay reagent were obtained from Promega Corporation (Madison, WI, USA). HepG2 and HepG2-NF-κB-Luc cells were provided by Prof. Y.C. Cheng (Department of Pharmacology, Yale University School of Medicine, USA). Cells cultured in Minimum Essential Media containing 10% fetal bovine serum were incubated at 37°C/5% CO_2_ and passaged three times a week.

### IKKβ enzymatic activity

IKKβ activity was determined using the ELISA-based (K-LISA™) IKKβ Inhibitor Screening Kit according to the manufacturer’s instructions. The GST-IκBα 50-amino acid peptide that includes the Ser32 and Ser36 IKKβ phosphorylation sites was used as a substrate and was incubated for 30 min at 30°C with human recombinant IKKβ in the presence of DMSO vehicle or different concentrations of **1** in a glutathione-coated 96-well plate. The phosphorylated GST-IκBα substrate was subsequently detected using anti-phospho-IκBα (Ser32/Ser36) antibody and a horseradish peroxidase-conjugated secondary antibody. The samples were finally incubated with TMB solution, and the color development was monitored at 450 nm on a plate reader (Bio-Rad).

### NF-κB transactivation activity

Exponentially growing HepG2-NF-κB-Luc cells were seeded overnight at 1 × 10^4^ cells/well in a 48-well plate. On the next day, the cells were pre-incubated with the indicated concentrations of **1** for 1 h before stimulation by 5 ng/mL of TNF-α for an additional 3 h. Passive lysis buffer (50 μL) was added to each well and the plate was incubated for 15 min with shaking. A 20 μL aliquot from each well was mixed with 70 μL luciferase assay reagent in a 96-well white plate. The transcriptional activity was determined by measuring the activity of firefly luciferase in a multi-well plate luminometer (Fusion α-FP, Perkin-Elmer).

### Molecular modeling

A natural product or natural product-like chemical library containing over 90,000 compounds was screened *in silico*. Molecular docking was performed by using the ICM-Pro 3.6-1d program (Molsoft). According to the ICM method, the molecular system was described by using internal coordinates as variables. Energy calculations were based on the ECEPP/3 force field with a distance-dependent dielectric constant. The biased probability Monte Carlo (BPMC) minimization procedure was used for global energy optimization. The BPMC global-energy-optimization method consists of 1) a random conformation change of the free variables according to a predefined continuous probability distribution; 2) local-energy minimization of analytical differentiable terms; 3) calculation of the complete energy including nondifferentiable terms such as entropy and solvation energy; 4) acceptance or rejection of the total energy based on the Metropolis criterion and return to step (1). The binding between the small molecules and NAE-NEDD8 were evaluated with a full-atom ICM ligand binding score from a multireceptor screening benchmark as a compromise between approximated Gibbs free energy of binding and numerical errors. The score was calculated by:

$$\begin{array}{l} S_{\mathrm{bind}}=E_{\mathrm{int}}+\mathrm{T\Delta}S_{\mathrm{Tor}}+E_{\mathrm{vw}}+\alpha_{1}E_{\mathrm{el}}+\alpha_{2}E_{\mathrm{hb}}+\alpha_{3}E_{\mathrm{hp}}\\ \;+\alpha_{4}E_{\mathrm{sf}} \end{array}$$

where *E*_vw_, *E*_el_, E_hb_, *E*_hp_, and *E*_sf_ are Van der Waals, electrostatic, hydrogen bonding, and nonpolar and polar atom solvation energy differences between bound and unbound states, respectively. *E*_int_ is the ligand internal strain, Δ*S*_Tor_ is its conformational entropy loss upon binding, and *T* = 300 K, and α_i_ are ligand- and receptor independent constants. The initial model of IKKβ was built from the X-ray crystal structure of the Inhibitor of kappaB kinase beta (PDB: 3RZF) according to a previously reported procedure. Hydrogen and missing heavy atoms were added to the receptor structure followed by local minimization by using the conjugate gradient algorithm and analytical derivatives in the internal coordinates. In the docking analysis, the binding site was assigned across the entire structure of the protein complex. Each compound was assigned the MMFF force field atom types and charges and was then subjected to Cartesian minimization. The ICM docking was performed to find the most favorable orientation. The resulting trajectories of the complex between the small molecules and protein complex were energy minimized, and the interaction energies were computed. Each compound was docked three times and the minimum of the three scores was used. The 12 highest scoring compounds were utilized for biological testing without further selection. The crystal structures of PAK4 (4APP), PKCα (3IW4), CAMK2α (2VZ6) and JAK2 (3IOK) were also prepared and compound **1** was docked to these molecular models individually using the aforementioned procedures.

## Competing interests

The authors declare that they have no competing interests.

## Authors’ contributions

C.-H. Leung and D.-L. Ma conceived the study, designed the experiments and performed the *in silico* high-throughput screening. Y.-W. Li and D. S.-H. Chan analyzed the experimental results, performed the experiments and wrote the manuscript. C.-H. Leung, D.-L. Ma and W.-F. Fong analyzed the experimental results and edited the manuscript. All authors have read and approved the final manuscript.

## Pre-publication history

The pre-publication history for this paper can be accessed here:

http://www.biomedcentral.com/2050-6511/14/3/prepub

## Supplementary Material

Additional file 1 **Figure S1.** Chemical structures of the 11 other high-scoring compounds selected for preliminary biological evaluation. **Figure S2.** Preliminary experimental screening of the 12 compounds on inhibition of cellular IKKβ mediated NF-κB activity. **Table S1.** Chemical name and structures of benzoic acid derivatives reported to target the NF-κB signaling pathway. **Table S2.** Lowest-energy binding pose of the 11 other compounds with the ATP binding site in the KD domain of IKKβ. **Table S3.** Binding poses and ICM docking energies of compound 1 to other four kinases. The reference compounds are displayed in cyan. Click here for file
