# Supplementary material for: Hit identification of IKKβ natural product inhibitor
Source: BMC Pharmacol Toxicol. 2013 Jan 7;14:3. doi: 10.1186/2050-6511-14-3 (PMC3583241; doi:10.1186/2050-6511-14-3)
Supplement: Additional file 1 — Figure S1. Chemical structures of the 11 other high-scoring compounds selected for preliminary biological evaluation. Figure S2. Preliminary experimental screening of the 12 compounds on inhibition of cellular IKKβ mediated NF-κB activity. Table S1. Chemical name and structures of benzoic acid derivatives reported to target the NF-κB signaling pathway. Table S2. Lowest-energy binding pose of the 11 other compounds with the ATP binding site in the KD domain of IKKβ. Table S3. Binding poses and ICM docking energies of compound 1 to other four kinases. The reference compounds are displayed in cyan. [file 2050-6511-14-3-S1.docx]

# Hit Identification of IKKβ Natural Product Inhibitor

### Chung-Hang Leung^1§^ Daniel Shiu-Hin Chan,^2^ Ying-Wei Li,^3^ Wang-Fun Fong,^3^ and Dik-Lung Ma^2§^

^1^ State Key Laboratory of Quality Research in Chinese Medicine, Institute of Chinese Medical Sciences, University of Macau, Macao, China

^2^ Department of Chemistry, Hong Kong Baptist University, Kowloon Tong, Hong Kong

^3^ Centre for Cancer and Inflammation Research, School of Chinese Medicine, Hong Kong Baptist University, Kowloon Tong, Hong Kong

^§^ Corresponding authors

Email addresses:

Chung-Hang Leung: duncanleung@umac.mo

Dik-Lung Ma: [edmondma@hkbu.edu.hk](mailto:edmondma@hkbu.edu.hk)

**-Figure S1 – Chemical structures of the 11 other high-scoring compounds selected for preliminary biological evaluation**

**

**

**Figure S2 – Preliminary experimental screening of the 12 compounds on inhibition of cellular IKKβ mediated NF-κB activity**

**
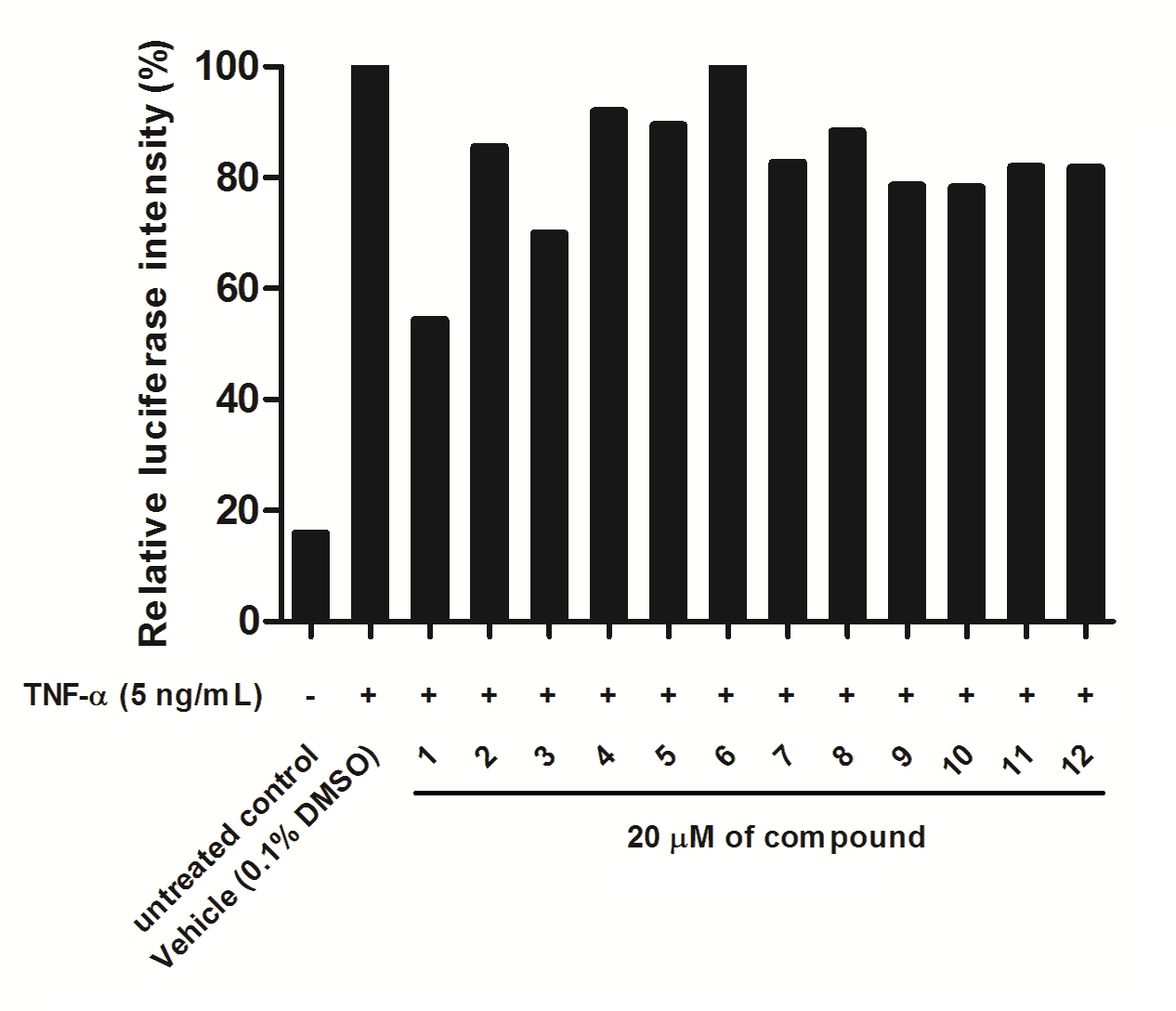
**

HepG2 cells stably transfected with a NF-kB luciferase reporter were treated with 20 µM of compound or vehicle (0.1% DMSO) for 1 h and stimulated with 5 ng/mL TNF-α for an additional 3 h. The luciferase activity of the cell lysates was determined using a luminometer.

**Table S1 – Chemical name and structures of benzoic acid derivatives reported to target the NF-κB signaling pathway**

| **Name** | **Chemical structure** | **Activities** | **Pharma-cological targets** |
| --- | --- | --- | --- |
| (*E*)-3-acetyl-6-(3,7-dimethylocta-2,6-dienyloxy)-2,4-dihydroxybenzoic acid |  | Suppression of fMetLeu-Phe (fMLP)-induced superoxide anion generation and elastase release by human neutrophils | N.A. |
| Antrocamphin A |  | downregulating the iNOS and COX-2 expression at both transcriptional and translational level by blocking the nuclear translocation of NF-κB | NF-κB |
| GS143 |  | Blocking the ubiquitylation of IκBα by SCF^βTrCPI^, thus preventing the activation of NF-κB | IκBα |

**Table S2 – Lowest-energy binding pose of the 11 other compounds with the ATP binding site in the KD domain of IKKβ**

| ZINC code | 2D structure | Lowest-energy binding pose |
| --- | --- | --- |
| ZINC02136133 |  | 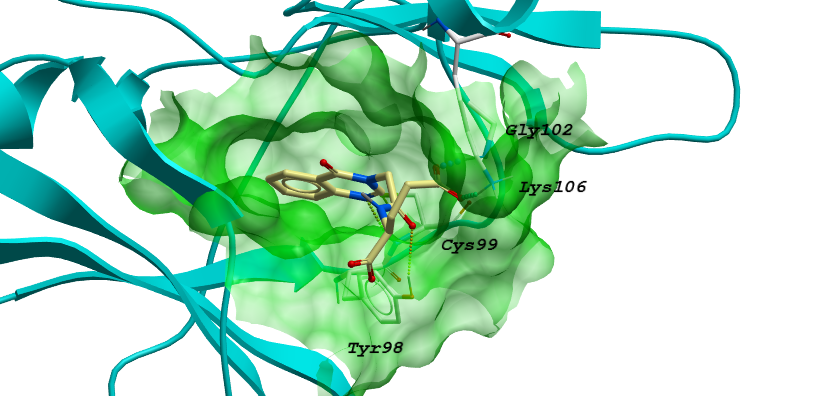 |
| ZINC02102156 |  | 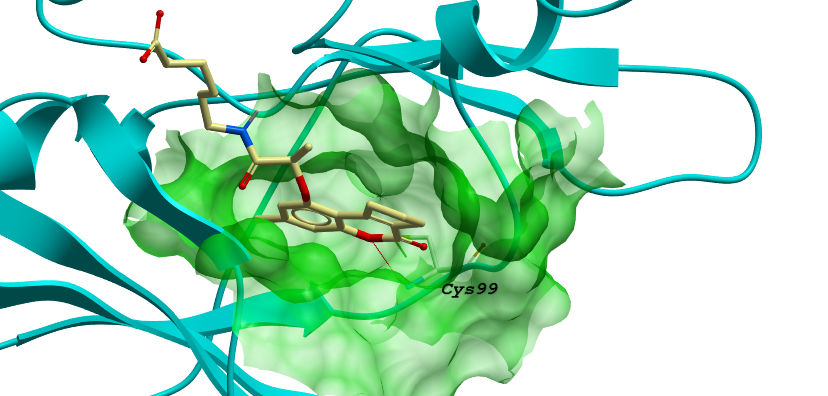 |
| ZINC01322850 |  | 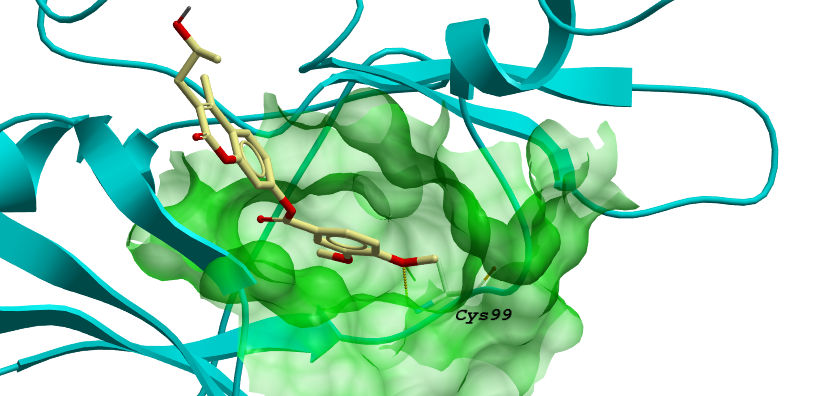 |
| ZINC07465287 |  | 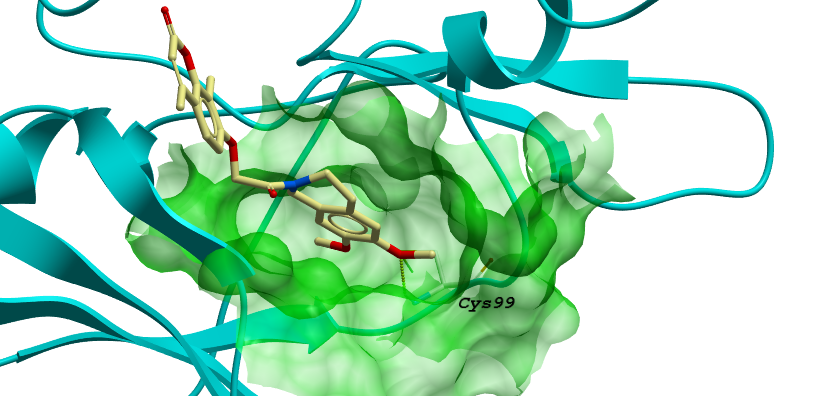 |
| ZINC12893150 |  | 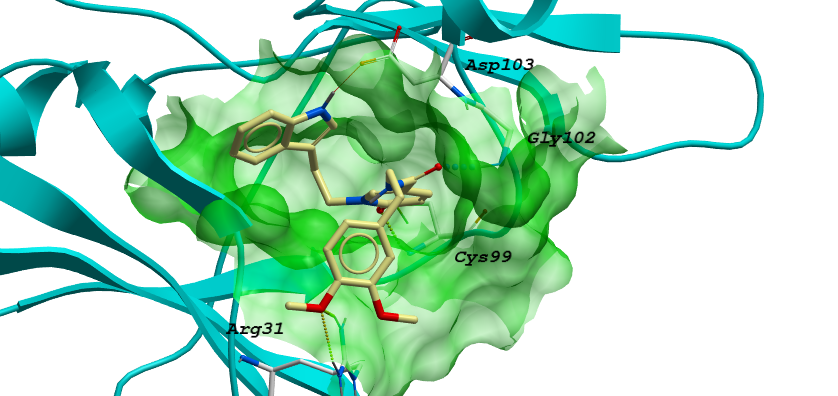 |
| ZINC02107060 |  | 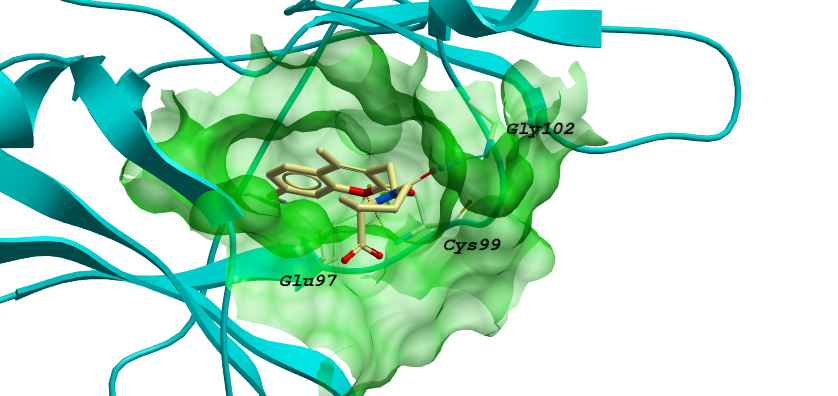 |
| ZINC02131319 |  | 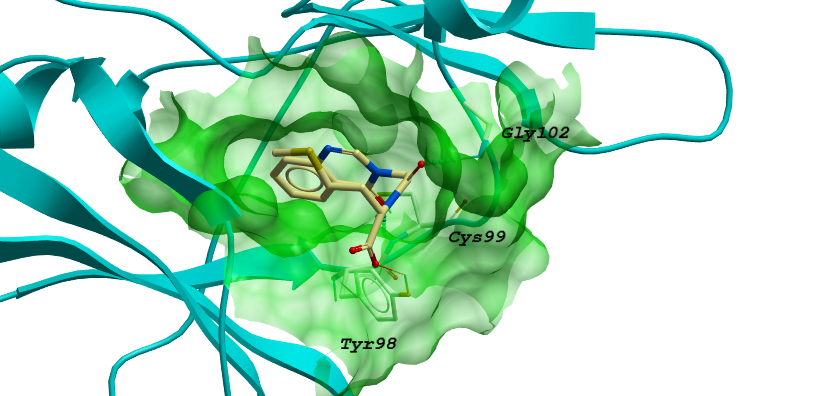 |
| ZINC08878184 |  | 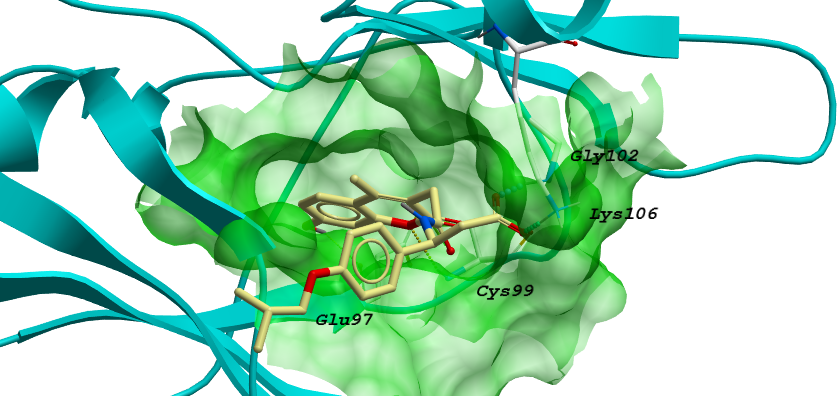 |
| ZINC08739362 |  | 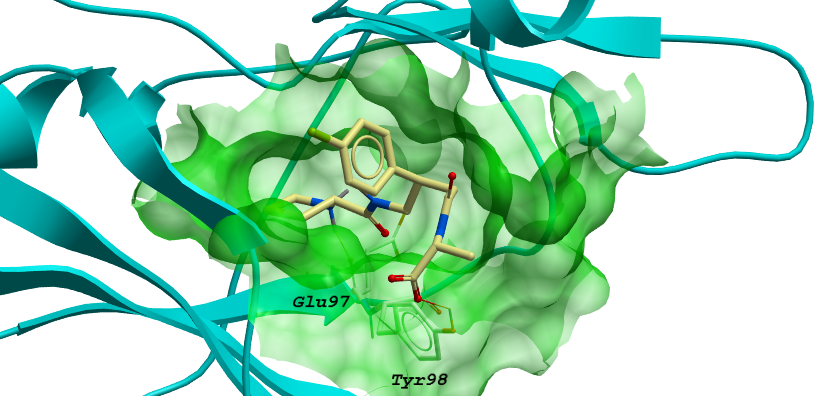 |
| ZINC02151090 |  | 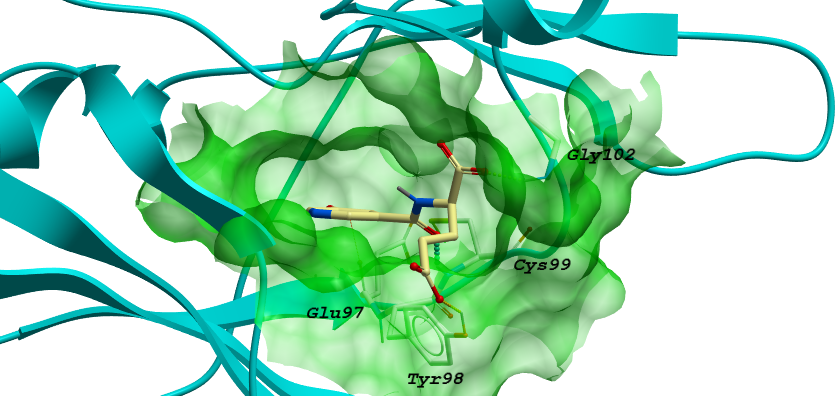 |
| ZINC12285473 |  | 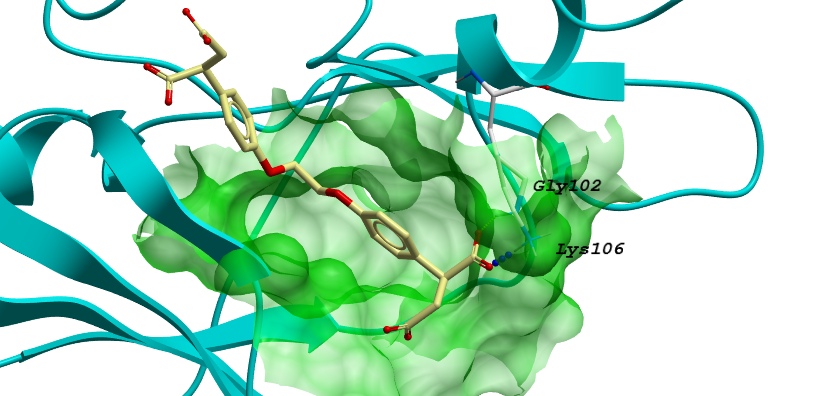 |

**Table S3 – Binding poses and ICM docking energies of compound 1 to other four kinases. The reference compounds are displayed in cyan.**

| Kinase | PDB | ICM energy (kcal/mol) | Lowest-energy binding pose |
| --- | --- | --- | --- |
| PKCα | 3IW4 | -4.6 | 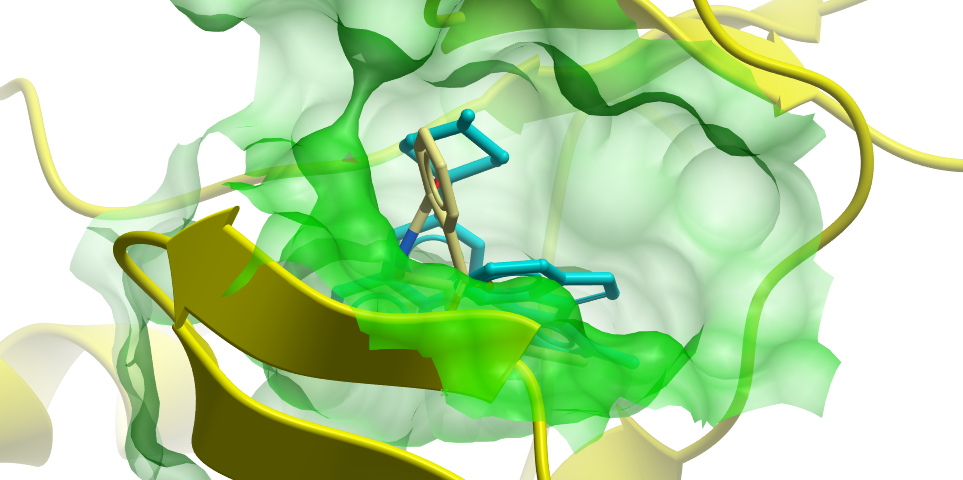 |
| PAK4 | 4APP | -9.2 | 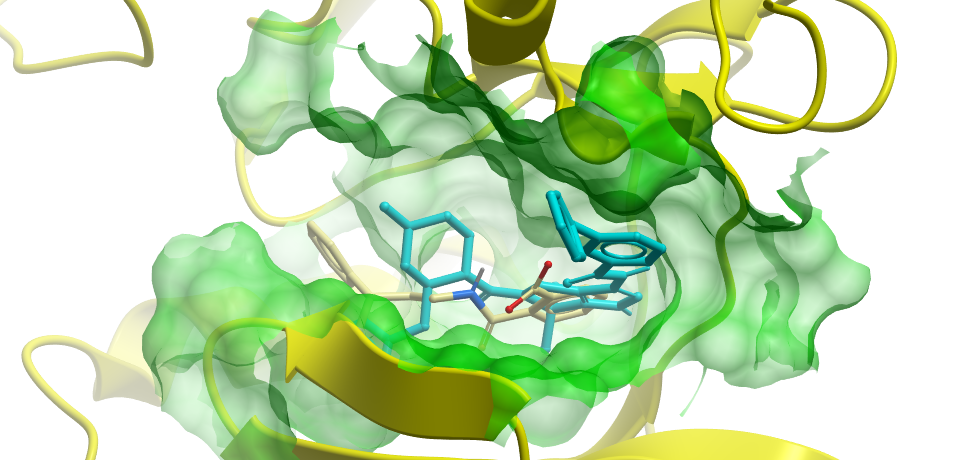 |
| CaMK2α | 2VZ6 | -16.0 | 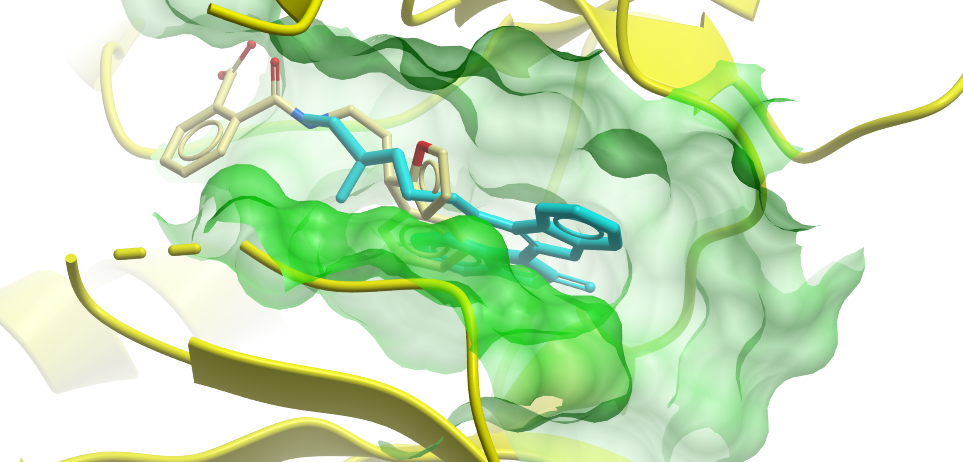 |
| JAK2 | 3IOK | -12.4 | 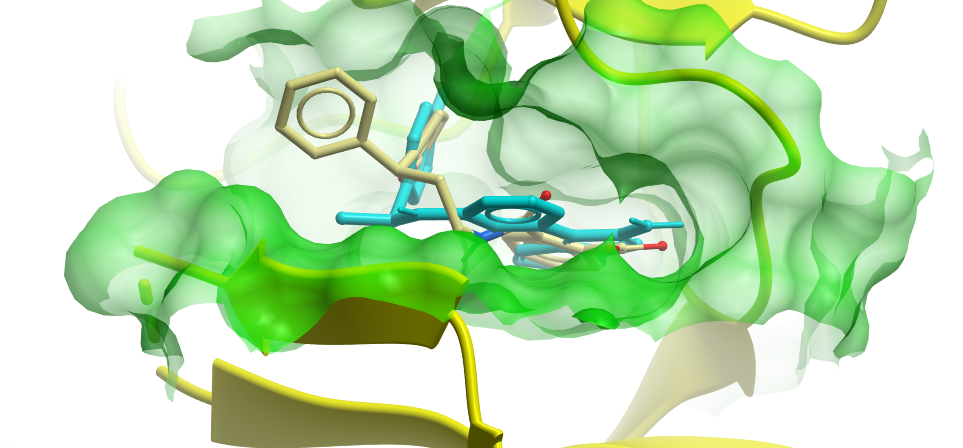 |
